# A new approach to varietal identification in plants by microsatellite high resolution melting analysis: application to the verification of grapevine and olive cultivars

**DOI:** 10.1186/1746-4811-4-8

**Published:** 2008-05-19

**Authors:** John F Mackay, Christopher D Wright, Roderick G Bonfiglioli

**Affiliations:** 1 Linnaeus Laboratory, Gisborne, New Zealand; 2 Riversun Nursery Ltd, Gisborne, New Zealand

## Abstract

**Background:**

Microsatellites are popular molecular markers in many plant species due to their stable and highly polymorphic nature. A number of analysis methods have been described but analyses of these markers are typically performed on cumbersome polyacrylamide gels or more conveniently by capillary electrophoresis on automated sequencers. However post-PCR handling steps are still required. High resolution melting can now combine detailed sequence analysis with the closed-tube benefits of real-time PCR and is described here as a novel way to verify the identity of plant varieties such as grapevine and olive.

**Results:**

DNA melting profiles for various plant variety and rootstock samples were compared to profiles for certified reference samples. Two closely related grapevine rootstocks differing by as little as a single di-nucleotide repeat could be rapidly differentiated while there was high reproducibility of melting profiles for identical cultivars.

**Conclusion:**

This novel microsatellite analysis method allows high sample throughput with greatly reduced time to results for varietal certification and is amenable to other microsatellite analyses.

## Background

Certification of grapevine scion and rootstock varieties is becoming an increasingly important issue in viticulture. Many rootstocks are derived from the same parentage – for example 5C, SO4, 125AA and 420A Mgt are all commonly used in New Zealand and are all derived from crosses between *Vitis berlandieri *and *Vitis riparia *while other popular rootstocks currently used are derived from *Vitis riparia *and *Vitis riparia *X *rupestris *parentage (Riparia Gloire du Montpellier, 3309C, 3306C, Schwarzmann and 101–14 Mgt). This use of limited genetic stock is not uncommon when compared with other countries [1] and means that cultivar determination based on ampelography (using visual characteristics of grapevines to determine identity) can be difficult at times due to the high similarities.

The use of microsatellite analysis has been well-characterised for grapevine scion [2,3] and rootstock varieties [3,4]. These markers are popular due to their highly stable and ubiquitous nature as well as co-dominant transmission of inheritance for parentage analysis. Various sets of microsatellites have been developed for the definitive resolution of scion and rootstock varieties [2,4]. Traditionally microsatellite analysis has relied on laborious polyacrylamide gels followed by silver staining or, more conveniently, fluorescent resolution on automated sequencers. Nonetheless this higher automation using sequencers still requires post-PCR handling and dilution steps as well as a fluorescently-labelled primer for each microsatellite which adds to the time and costs involved. For the analysis of amplified DNA, the use of real-time PCR is becoming increasingly common in viticulture – among other industries – for virus and pathogen identification [5,6] as well as rapid analysis of other reactions that require no further handling of the amplified DNA. This has led to decreased contamination risks and faster turnaround times in molecular diagnostics, as well as providing quantification opportunities for assessing pathogen levels.

Microsatellites and repeat sequence polymorphisms have been studied previously using real-time PCR chemistries such as hybridisation probes [7,8]. However in this instance, sequence specific probes are required for each microsatellite under analysis – again adding to the costs involved when studying a number of loci for certification purposes. In addition only the region under (or very near to) the probes may be analysed.

We investigated a more recent development in fluorescent analysis of PCR products; high resolution amplicon melting analysis, and applied this technique to the analysis of microsatellite markers in a number of rootstock and grapevine scion varieties commonly grown in New Zealand. High resolution melting analysis has been used primarily for the discovery and genotyping of single nucleotide polymorphisms (SNPs) [9], but it has also been used for precise amplicon verification [10] and sequence matching applications such as HLA identity [11]. The method involves the gradual denaturation (melting) of these PCR amplicons and detection of the subsequent subtle fluorescent changes by so-called 3^rd ^generation fluorescent double stranded DNA-binding dyes present in the amplification reaction [12]. Problems with SYBR Green melting curve analysis have been well described with the dye's preference for intercalating in GC-rich regions of amplicons and its tendency to translocate during melting phases [13,14]. This is due to its relatively low concentration in a PCR reaction to avoid amplification inhibition. Dyes for high resolution melting curve analysis are used at a higher 'saturating' concentration and thereby overcome these limitations. They include LC Green PLUS (Idaho Technologies Ltd), EvaGreen (Biotium), SYTO9 (Invitrogen) and ResoLight dye (Roche Applied Science).

Samples are amplified in the presence of these newer dyes in a similar manner to SYBR Green-based real-time PCR and following the cycling process, the PCR amplicons are slowly melted with higher data acquisition levels than with SYBR Green applications. Using an instrument with precise temperature control and homogeneity among the samples being analysed, slight sequence variation between samples can be detected by denaturation at the DNA polymorphism and subsequent fluorescent decreases caused by the dissociation of the double stranded DNA-specific dye. As these dyes are able to be used at higher concentrations than SYBR Green I and are therefore more sensitive to sequence variation during melting, these fluorescent changes are reflected in the differing shapes of the melting curves. In this work we investigated whether the precision and reproducibility of the high resolution melting curves could be used to verify microsatellite identity of highly heterozygous plant varieties such as grapevine by comparing the melting curves of microsatellite amplicons for a suspected variety and a known reference sample of the variety.

## Results and Discussion

### Reaction conditions

Initially, the amount of DNA per reaction was not standardised before amplification – up to 10-fold variation in template concentrations may be used in high resolution melting reactions [15] due to the lower dependence of Tm on template concentration. In addition, fluorescent yield variation that can be due to template concentration variation is accounted for in the normalisation steps after amplification. However increased reproducibility of melting curves was observed by first quantifying and diluting the samples to a uniform 20 ng.μl^-1 ^prior to amplification. This was likely due to the slight polysaccharide variation (and/or other inhibitors) of the undiluted grapevine samples and therefore dilution of samples to a standard template concentration removed this variation as seen by the similar amplification crossing points of the samples – the crossing point being the threshold cycle number near where the reaction enters the exponential phase of amplification (data not shown).

**Figure 1:**
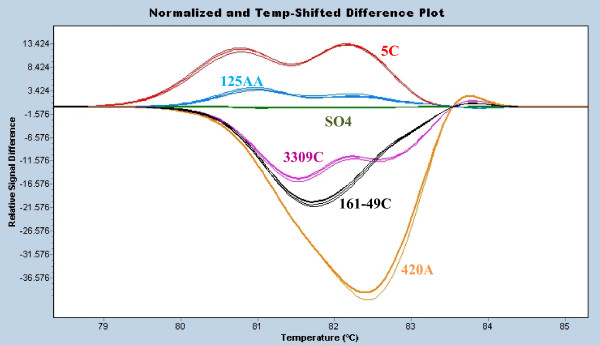
**Rootstock differentiation**. Difference plot of the ZAG62 microsatellite for 6 closely related rootstocks derived from *Vitis berlandieri *crosses and *Vitis riparia *crosses: 5C, SO4, 125AA, 161-49C, 3309C and 420A. 4 DNA extractions for each rootstock were amplified in order to show the reproducibility of melting curves.

We developed a universal cycling protocol of touchdown PCR to allow all tested grapevine microsatellites for a sample to be amplified and analysed within the same run. This, together with simple analysis steps, permitted the matching of queried samples with reference cultivars within 90 minutes from start of amplification – or less than 3 hours from sample to result. This represents an order of magnitude increase in speed and decreased time to results compared with other typically used analysis methods for microsatellite amplicons. In many cases, the melting plots of a single microsatellite allowed the differentiation of a number of varieties – particularly in the case of common rootstocks used in the New Zealand industry (Figure 1).

### Reproducibility

**Figure 2:**
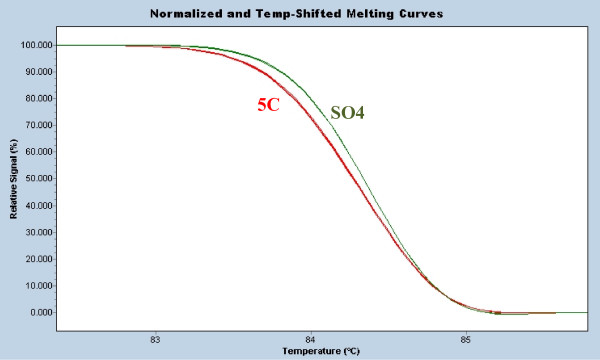
**Detecting a single di-nucleotide repeat difference**. Two closely-related rootstocks (5C and SO4) share one allele size (259 bp) and differ by a single CT repeat at the other allele (236 bp for 5C and 234 bp for SO4) for microsatellite locus VVMD32. Five individual extractions for 5C and SO4 show the differentiation of these two rootstocks

While replicates are often performed for high resolution melting analysis due to the technique being highly dependent on salt and buffer conditions in a reaction, our routine analyses were usually performed on individual DNA extractions compared against extractions of certified standards. Nonetheless, high reproducibility of melting curves was observed between individual samples and reference standards run singly. This high level of reproducibility was also shown by the analysis of multiple individual DNA extractions for the same variety (Figures 1, 2). The melting point (Tm) standard deviation for each genotype typically ranged from 0.009 to 0.06°C. This correlates well with other reproducibility studies performed for high resolution melting performance [16] The reference sample identities were previously verified through ampelography or sourced from reference centres (e.g. Etablissement National Technique pour l'Amélioration de la Viticulture (ENTAV)).

### Sensitivity

In order to test the differentiation ability of the microsatellite high resolution melting, we examined two rootstocks that have been historically confused [17]. Previous analysis of rootstocks 5C and SO4 at microsatellite VVMD32 showed they both shared an allele and differed by 2 bp (i.e. a single CT di-nucleotide repeat) at the other allele [3]. High resolution amplicon analysis was able to clearly differentiate these samples (Figure 2) with high reproducibility observed between the five samples of each rootstock variety as indicated previously.

### Discrepant samples

**Figure 3:**
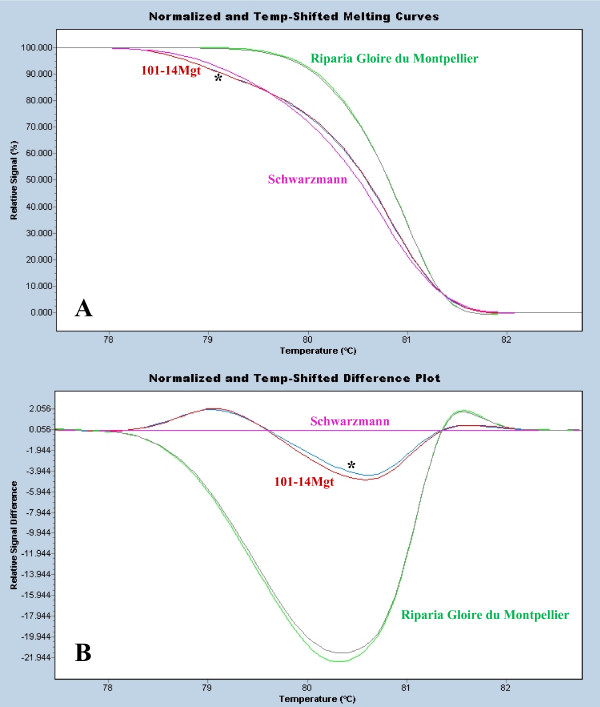
**Rapid detection of incorrectly identified rootstock**. A rootstock sample (*) stated as Schwarzmann matched 101–14 Mgt at microsatellite VVMD5 when melting curves were adjusted along the temperature axis (A). The differences in the melting curves are highlighted in the difference plot (B) using the plot for Schwarzmann as the baseline reference (horizontal line).

As mentioned, even for comparison of samples with similar allele sizes, a distinct and reproducible difference could be observed permitting the rapid resolution of incorrectly identified varieties (Figure 3). A rootstock sample labelled as Schwarzmann was submitted for certification testing. It was found to have a melting curve for microsatellite VVMD5 identical to that of a similar parentage rootstock: 101–14 Mgt. This discrepancy was highlighted in the difference plot (Figure 3B) and the sample was confirmed as 101–14 Mgt using other microsatellite markers (data not shown) as well as ampelography. Rootstocks Schwarzmann and 101–14 Mgt are both derived *Vitis riparia *X *rupestris *crosses, have one allele in common for microsatellite VVMD5 and differ by 3 bp at the other allele according to a previous analysis [4]. However the distinctive melting profiles for each rootstock allowed the rapid resolution of the incorrectly classified sample.

### Analysis means

**Figure 4:**
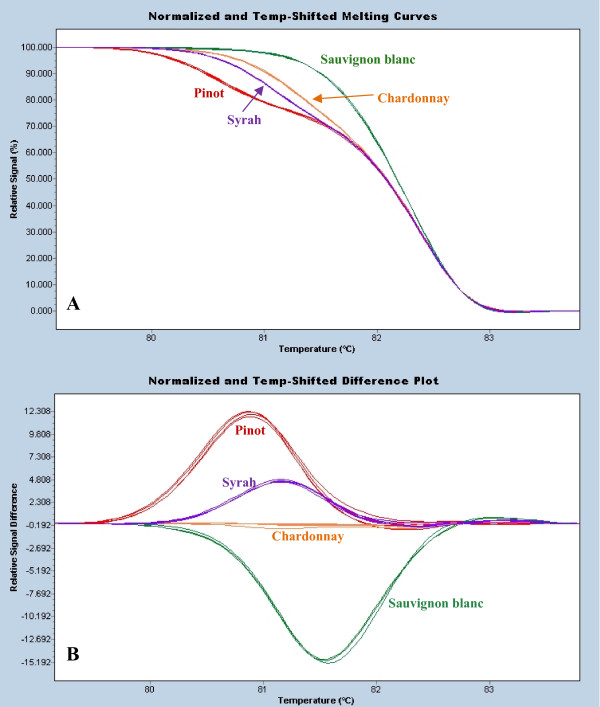
**Scion variety differentiation**. Temperature-shifted melting curves (A) and difference plots (B) for the differentiation of scion varieties for Pinot (allele sizes 238:244 bp), Syrah (244:250 bp), Sauvignon blanc (244:246 bp) and Chardonnay (242:244 bp) at microsatellite locus ZAG79. Duplicate samples for two clones of each variety were extracted and amplified. Allele sizes from [28].

**Figure 5:**
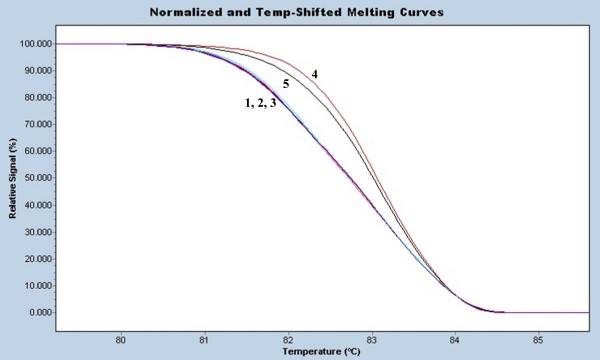
**Addition of reference cultivar to query samples**. Reference DNA was added to query samples and amplified together with the reference and query samples amplified individually. (1) reference Riesling (2) query Riesling 1 (3) query Riesling 1/reference Riesling mixed sample (4) query Riesling 2 (5) query Riesling 2/reference Riesling mixed sample.

**Figure 6:**
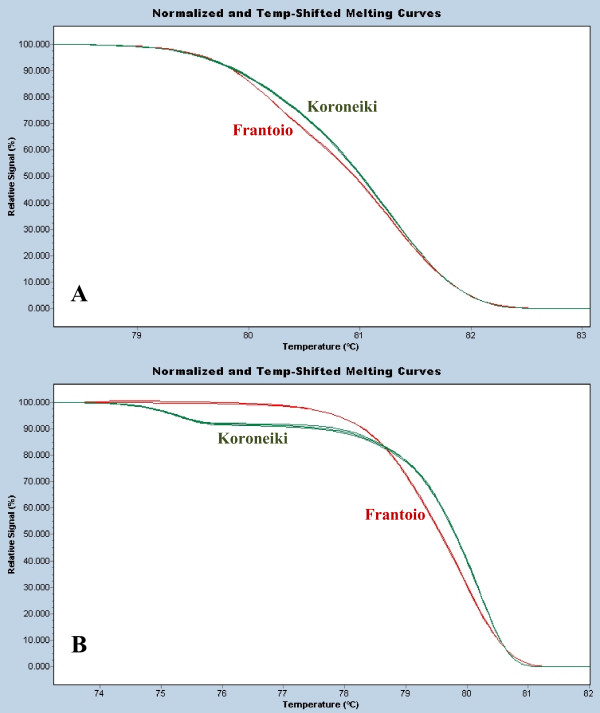
**Analysis of Olive cultivars**. Five DNA extractions for each olive cultivar 'Frantoio' and 'Koroneiki' showing distinctive melting profiles with the microsatellites DCA-3 (A) and UDO-12 (B).

There are a number of convenient analysis methods for high resolution melting – either temperature-shifted melting curves (Figures 2, 3a, 4a, 5 and 6) or difference plots (Figures 1, 3b and 4b) which serve to highlight differences between individual curves relative to one of the sample melting curves plotted as a baseline. For individual reactions we found that the temperature-shifted melting curves were sufficient to rapidly determine a match although difference plots also proved useful for clustering larger numbers of samples and allowing more distinct separation of varieties (Figure 4).

### Reference DNA addition

Further resolution of genotypes in SNP typing may be gained by the addition of a reference DNA of known genotype to the sample under query prior to amplification [11,18]. Typically, if the query sample and reference have identical sequences then there is no difference in melting curves between the mixed query/reference sample and the reference sample alone, due to lack of heteroduplex formation. If there is a sequence difference then heteroduplex formation between the query and reference amplicons will generate a melting curve able to be better distinguished from the reference and query samples amplified individually. Our preliminary trials of this reference addition approach in this microsatellite analysis method proved useful for further confirmation of matching between sample and reference (i.e. no variation was observed between query sample, reference sample and query/reference mixed sample as the case with melting curves 1, 2 and 3 in Figure 5). However there was no increase in resolution of non-matching query and reference mixed sample (melting curve 5) over comparing the query (melting curve 4) and reference (melting curve 1) amplified individually. We surmised this was likely due to the heteroduplex formation already formed between the two alleles of the microsatellite locus in the individual sample. The addition of a reference DNA will only lead to a more complex heteroduplex mix in this instance. However this approach may prove useful for microsatellite analyses in the case of null or homozygous alleles where only one amplicon size may be generated, as in the case of rootstock St. George at the VVS2 allele [4].

The varying 'bulges' or looping out of the non-hybridised repeats of the longer alleles in the heteroduplexes characterise the specific melting curves. This may be due to nearest neighbour thermodynamics with the non-hybridised base sequence affecting the stability and melting of the perfectly matched bases adjacent to the bulges [19], the physical influence of the loop size influencing the secondary structure of the DNA [20] and thus the amplicon melting, or a combination of both and more.

There is a drawback to this method compared with other methods, at least at time of writing. This high resolution melting method currently cannot be used to establish a database for unknown cultivar determination, as the melting curves cannot be saved for external reference. Therefore the method can only be used for comparison purposes at this time; comparing test samples with reference samples of the suspected variety/varieties. However high resolution melting should prove useful as a preliminary screen with any subsequent discrepant samples undergoing capillary electrophoresis for database matches. A database utility of melting plots is envisaged in the future for this methodology.

### Further applications

In order to investigate wider applicability of this method for microsatellite analysis, we have also investigated olive cultivars. Using the methods described here, cultivars Frantoio and Koroneiki could be easily differentiated at two tested microsatellite loci (Figure 6). In addition, early work has shown the differentiation of pine tree cultivars (data not shown). We see this method therefore as a general purpose method for comparison of microsatellites in query samples to known reference identifier samples.

## Conclusion

High resolution melting is a recent development on product verification via dye melting from amplicons. However compared to typical amplicon melting using SYBR Green dye, the method provides significant benefits in sequence specificity and resolution sensitivity. Its speed and closed-tube nature provide similar benefits as with other real-time PCR applications; in this instance higher sample throughput is achieved along with ease of microsatellite comparisons for our grapevine certification and other crop programs. This new analysis method is amenable to other microsatellites being investigated especially for those laboratories without immediate access to a capillary sequencer.

## Methods

### DNA extraction

100–150 mg young unexpanded leaf material from each sample was homogenised in 1.5 ml AP1 buffer from the DNeasy^® ^Plant Mini kit (Qiagen) using a homogeniser mounted on a drill press. 400 μl of the homogenate was further processed and genomic DNA extracted using the DNeasy^® ^Plant Mini kit according to the manufacturer's protocol. DNA was eluted in a final volume of 200 μl, quantified on a Nanodrop ND-1000 (Nanodrop Technologies) and diluted to a concentration of 20 ng.μl^-1 ^in PCR-grade water. DNA from olive varieties Frantoio and Koroneiki were extracted using a modified CTAB protocol [21], quantified and diluted as for grapevine samples.

### Microsatellite amplification

We analysed the following microsatellites for grapevine: VVS2 [22], VVMD5, 7 [23], VVMD24, 27, 32, 36 [24], ZAG62 and ZAG79 [25]. These are di-nucleotide repeat sequence motifs with allele sizes ranging from 129 bp (the smallest with VVS2) through to 315 bp (the largest for VVMD36). Amplification and high resolution melting analysis were performed on the LightCycler^® ^480 system (Roche Applied Science) using 10 or 20 μl reactions in 96-well multiwell plates (Roche Applied Science). PCR reactions consisted of 1X LightCycler^® ^480 High Resolution Melting Master (Roche Applied Science); supplemented with 2 mM MgCl_2_, 0.25 μM each primer and 50 ng genomic DNA. Samples with added reference DNA consisted of 7 ng certified reference DNA and 43 ng query sample DNA – i.e. a 15% proportion of reference DNA as used in [18].

The cycling program for all tested microsatellites consisted of a touchdown protocol: 5 minutes initial denaturation followed by 45 cycles of denaturation at 96°C (8 seconds), annealing from 56°C to 54°C (8 seconds) and extension at 72°C (12 seconds). The annealing temperature decreased in subsequent cycles by 0.5°C per cycle after the first 56°C annealing step to 54°C. The amplification cycles were immediately followed by the high resolution melting steps of 95°C for 1 minute, cooling to 40°C for 1 minute, raising the temperature to 65°C and then raising the temperature to 95°C with 25 fluorescent acquisitions per degree Celsius at this step. Olives were analysed with microsatellites DCA-3, DCA-16 [26] and UDO-12 [27] and were amplified in a similar touchdown protocol but with annealing temperatures decreasing from 60°C to 55°C.

### High resolution melting curve analysis

After the verification of robust amplification curves and the presence of a specific melting peak for the microsatellite amplicon, the melting curve stage was further analysed with the gene scanning software module on the LightCycler^® ^480 instrument. Absolute fluorescent levels pre- and post-melt were normalised to 100% and 0% respectively to account for any varying fluorescent yields from different reactions. As high resolution melting analysis is based on the shape of the melt curves rather than absolute melting Tm derived from the negative derivative (as traditionally seen with SYBR Green amplifications), the melt curves are then normalised along the temperature axis ('temperature shifting') to permit easy differentiation of DNA curves (more information on these analysis algorithms can be found in [15]). Query samples were compared with certified reference plots for each variety.

## Competing interests

The authors declare that they have no competing interests.

## Authors' contributions

JFM conceived of the study, designed and performed the experiments and wrote the manuscript. CDW sourced a number of the reference materials used, contributed ampelographic expertise, resolved discrepant samples and reviewed the manuscript. RGB sourced several of the reference materials from international collections, oversaw the project and revised the manuscript. All authors read and approved the final manuscript.
